# The relationship between living alone or not and depressive symptoms in older adults: a parallel mediation effect of sleep quality and anxiety

**DOI:** 10.1186/s12877-023-04161-0

**Published:** 2023-08-22

**Authors:** Mina Huang, Kun Liu, Chunguang Liang, Yongzhu Wang, Zhanpeng Guo

**Affiliations:** 1 Department of Nursing, The Jinzhou Medical University, Jinzhou, China; 2 Department of Medical College, The Jinzhou Medical University, Jinzhou, China; 3 Department of Orthopedics, The First Affiliated Hospital of Jinzhou Medical University, No.2, Section 5, Renmin Street, Jinzhou, Liaoning 121001 China

**Keywords:** Living alone, Depressive symptoms, Sleep quality, Anxiety, Older adults, Living arrangements, CLHLS

## Abstract

**Background:**

With modernization and ageing in China, the population of older adults living alone is increasing. Living alone may be a potential risk factor for depressive symptoms. However, no parallel mediation model analysis has investigated the mediating factors for living alone or not (living arrangements) and depressive symptoms.

**Methods:**

This cross-sectional study included a total number of 10,980 participants from the Chinese Longitudinal Healthy Longevity Survey (CLHLS), 1699 of whom lived alone and 9281 of whom did not live alone. Binary logistic regression and parallel mediation effect model were used to explore the relationship between living alone or not and depressive symptoms and possible mediation effects. Bootstrap analysis was used to examine the mediation effect of living alone or not on depressive symptoms.

**Results:**

Compared to the participants who were not living alone, the living alone group had a higher rate of depressive symptoms. The binary logistic regression showed that after adjusting for other covariates, the risk of depressive symptoms was approximately 0.21 times higher for living alone compared to not living alone (OR = 1.21, 95% CI: 1.06, 1.37). Further, the results of the bootstrap analysis supported the partial mediating role of sleep quality and anxiety. Mediation analysis revealed that sleep quality and anxiety partially mediate the relationship between living alone and depressive symptoms (β = 0.008, 95% CI [0.003, 0.014]; β = 0.015, 95% CI [0.008, 0.024], respectively).

**Conclusions:**

Sleep quality and anxiety were identified as partially parallel mediators between living alone or not and depressive symptoms. Older adults living alone with poorer sleep quality and more pronounced anxiety were positively associated with higher levels of depressive symptoms. Older adults living alone should be encouraged to engage in social activities that may improve sleep quality, relieve anxiety, and improve feelings of loneliness caused by living alone. Meanwhile, older adults living alone should receive attention and support to alleviate their depressive symptoms.

**Supplementary Information:**

The online version contains supplementary material available at 10.1186/s12877-023-04161-0.

## Introduction

According to the results of the seventh national census in 2020, the number of Chinese over the age of 60 reached about 264.02 million, accounting for 18.70% of the total population [1]. Compared with the sixth national census, the proportion was above 5.44% points [2]. As the elderly population continues to grow, the health problems of older adults are increasing of public concern [3–6]. Due to various factors such as physical ageing, chronic diseases, declining social relationships, and economic status, older adults may have psychological problems related to negative emotions [7–9]. Depression is a prevalent mental health problem in older adults [10]. Depression mainly includes mood symptoms, neuropathic symptoms, and negative symptoms; depressed mood and lack of pleasure are the primary symptoms of depression [11]. It can lead to decreased interest in daily life activities, poor memory, etc. [12]. Research showed that associated factors with old-age depression include sex, chronic disease, cognitive impairment, functional impairment, etc. [13]. A systematic review evaluated the rate of depressive symptoms in Chinese older adults to be approximately 20.0% [14]. Therefore, research on depression in older adults has implications for identifying or improving depression in older adults.

With the modernization and demographic transition the traditional Chinese co-housing model is gradually changing [15]. The living arrangements of older adults in China have changed significantly [16]. The number of older adults who choose to live alone for active or passive reasons is increasing. Research demonstrated that older adults living alone have poorer health and mental health [17–19]. These unhealthy outcomes were regarded as cognitive decline, blood pressure, poorer physical health, anxiety and depression, etc. [20–23]. Older adults living alone felt more sad and hopeless [24], and empty-nest elderly have poorer physical health [25]. Research also indicated that older adults who lived alone were related to cognitive decline [26]. In addition, poor social network size was related to more obvious depressive symptoms in older adults living alone [27]. According to previous studies, we found that the relationship between living alone or not and depressive symptoms was obvious.

Sleep quality is another important issue in the lives of older adults living alone. The prevalence of sleep disorders in older adults was 10.4-62.1% [28]. Older adults and females were likely to have poor sleep quality [29]. Also, older adults living alone showed poorer sleep quality and female older adults who live alone had a higher poor sleep quality than those living with others [30]. Therefore, females, living alone and being older may likely have poorer sleep quality [31]. Scholars have pointed out that sleep quality was associated with depression, especially in elderly women living alone [32]. Other studies have also demonstrated the impact of sleep quality on depression [33–35]. Meanwhile, sleep quality played a mediating role between chronic diseases and depressive symptoms in the elderly [36]. Also, sleep quality served as a mediator between cognitive decline and depression in older adults [37]. Therefore, sleep quality may mediate depressive symptoms in older adults.

Anxiety, which is defined as a negative emotional state characterized by nervousness or anxiety, is another predominant mental health problem among older adults [38]. Anxiety symptoms were associated with depression in older adults living alone [39]. Previous study has shown that anxiety symptoms could mediate the association between loneliness and cognitive function among older adults [40]. However, no related research has addressed the possible mediating role of anxiety in the relationship between living alone or not and depressive symptoms. Therefore, we hypothesized that anxiety plays a role in the relationship between living alone or not and depressive symptoms.

Although previous researchers have shown the effects of depressive symptoms, sleep quality and anxiety in older adults living alone. Some studies have also explored the relationship between living arrangements and depression. Despite this, little research has been done on the mediating effects of sleep quality and anxiety between living alone or not and depressive symptoms. And it has not been investigated whether sleep quality and anxiety play a parallel mediating role in this effect. So this study aimed to investigate the relationship between living arrangements (living alone or not) and depressive symptoms in older adults and the parallel mediating role of sleep quality and anxiety in this relationship. We hypothesized that (1) the older adults living alone are more likely to have depressive symptoms; (2) sleep quality and anxiety could parallel mediate the relationship between living alone or not and depressive symptoms.

## Materials and methods

### Study design and participants

The data were derived from the Chinese Longitudinal Healthy Longevity Survey (CLHLS), which was a nationwide survey project of the Center for Healthy Aging and Development, National School of Development, Peking University [41]. The current study was the latest data of the CLHLS − 2018 wave. The data covered more than 500 sample sites in 22 of China’s 31 provinces, with more than 15,000 participants aged 65 and over [42].

The survey covered sociodemographic information, emotional characteristics, activities of daily living, health-related issues, economic status, etc. All data were obtained through face-to-face interviews. Participants were asked for sociodemographic information, including sex, age, residence, marital status, living arrangements and more. They were also asked about health-related issues, including self-reported health, sleep time, drinking, smoking and exercise habits, among other things. In addition, they were asked if they had chronic diseases. Economic status was also under investigation. Furthermore, the Center for Epidemiological Studies Depression Scale (CES-D-10) [43] and the Anxiety Disorder Scale (GAD-7) [44] assessed mental status, the Basic Activities of Daily Living (BADL) and Instrumental Activities of Daily Living (IADL) [45] were considered as assessments of the activities of daily living.

**Fig. 1:**
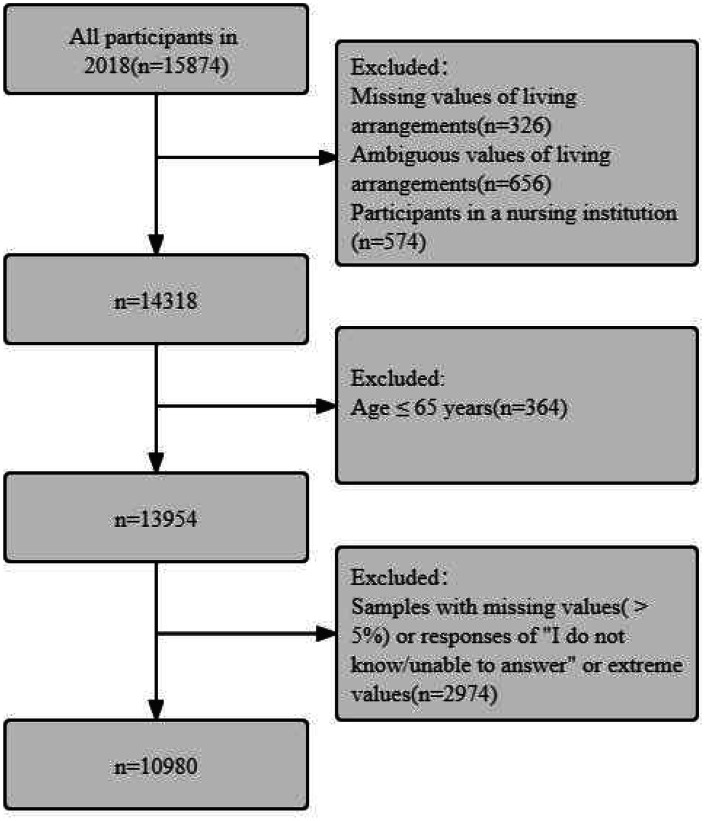
Flow diagram of how to select participants

This study used the CLHLS 2018 wave to investigate the depressive symptoms in older adults living alone or not and to analyze possible mediation effects. The inclusion criteria for this study were: (1) age > 65 years; (2) participants who were living with household member(s) and living alone. The exclusion criteria were: (1) participants who were living in a nursing institution; (2) among the variables of interest, samples with missing values(> 5%) or responses of “I don’t know/unable to answer” or extreme values. Mean imputation were used to replace missing values(< 5%). Finally, a total of 10,980 participants were included in this study. The detail of how to select participants was shown in Fig. 1.

### Measures

#### Dependent variables

Depressive symptoms were evaluated by the Center for Epidemiological Studies Depression Scale (CES-D-10) [43]. The depression scale (CES-D-10) consisted of ten items using a four-point metric as 0 for “never”, 1 for “sometimes or rarely”, 2 for “often” and 3 for “always”. The score of CES-D-10 ranged from 0 to 30, with higher scores indicating more pronounced depression. Individuals were considered to have depressive symptoms when they had a score of at least 10 [46]. Studies have shown that CES-D-10 has shown promising reliability among Chinese people [47]. The coefficient of Cronbach alpha for CES-D-10 in this study was 0.783. The internal consistency of the scale was reasonable.

#### Independent variables

Living arrangements (living alone or not) were evaluated using the question “Who do you live with?” which included the responses “with household members (not living alone)” and “living alone”.

#### Covariates

Covariates included sociodemographic characteristics, health-related issues, economic factors, activities of daily living and anxiety. The sociodemographic characteristics included age, gender, residence and marital status. Health-related issues included self-reported health (how do you feel about your health now?), sleep quality, social activities (do you take part in social activities?), sleep time (h)/day, smoking, drinking, exercises, number of chronic diseases (hypertension, dyslipidaemia, diabetes, cancer, heart attack, stroke, Parkinson’s disease, arthritis, etc.). Economic factors included sufficiency of living source (is all of the financial support sufficient to pay for daily expenses?), economic status (how do you rate your economic status compared with other local people?).

The anxiety disorder scale (GAD-7) was used to assess anxiety. GAD-7 measures the frequency of anxiety-related symptoms over the past two weeks and consists of seven items, each of which was answered by: 0 = never, 1 = a few days, 2 = more than half of the days, and 3 = almost every day. The total score ranged from 0 to 21. An individual with an anxiety score greater than 4 was defined as having anxiety [48]. It has been validated among older Chinese adults [49]. There was high internal consistency of the scale (Cronbach’s alpha = 0.919) in this study.

The basic activities of daily living (BADL) and instrumental activities of daily living (IADL) were used to evaluate activities of daily living. BADL included six items, such as bathing, dressing, using the toilet, eating, etc. Each item was scored from 1 to 3 (1 = don’t need help; 2 = partially need help; 3 = need help). There was a range of scores from 6 to 18. The higher the score, the greater the BADL dependence. The BADL was defined as “need help” when “partially need help and need help” were chosen for at least one item. A Cronbach alpha coefficient of 0.847 has been calculated for the BADL. IADL was about the instrumental activities which included eight items with each item scored from 1 to 3 (1 = complete independence; 2 = partial dependence; 3 = complete dependence) [50]. The IADL’s score ranged from 8 to 24. The higher the score, the greater the IADL dependence. The IADL was defined as “dependence” when “partially and complete dependence” were chosen for at least one item. The Cronbach alpha coefficient of the IADL was 0.945.

The details of the variable assignments were shown in Table 1.

**Table 1:** Basic characteristics and univariate analysis of the participants by depressive symptoms

Variables	Assignments	N (%)/Mean(SD)			χ^2^ /t	*P*
		Total (N = 10,980)	Depressive symptoms			
			No(N = 5094)	Yes(N = 5886)		
**Sociodemographic characteristics**						
**Gender**					80.657	< 0.001
Male	Male = 0	5134(46.8)	2616(51.4)	2518(42.8)		
Female	Female = 1	5846(53.2)	2478(48.6)	3368(57.2)		
**Age(years)**	Continuous measurement	83.63(10.85)	82.62(10.81)	84.51(10.81)	-9.181	< 0.001
**Residence**					60.301	< 0.001
City	City = 0	2500(22.8)	1330(26.1)	1170(19.9)		
Town/rural	Town/rural = 1	8480(77.2)	3764(73.9)	4716(80.1)		
**Marital status**					132.102	< 0.001
Married	Married = 0	5165(47.0)	2696(52.9)	2469(41.9)		
Others	Others = 1	5815(53.0)	2398(47.1)	3417(58.1)		
**Living arrangements**					46.034	< 0.001
Not living alone	Not living alone = 0	9281(84.5)	4434(87.0)	4847(82.3)		
Living alone	Living alone = 1	1699(15.5)	660(13.0)	1039(17.7)		
**health-related**						
**self-reported health**					752.509	< 0.001
Good	Good = 0	5321(48.5)	3185(62.5)	2136(36.3)		
poor	poor = 1	5659(51.5)	1909(37.5)	3750(63.7)		
**Sleep quality**					1216.429	< 0.001
Good	Good = 0	5825(53.1)	3612(70.9)	2213(37.6)		
poor	poor = 1	5155(46.9)	1482(29.1)	3673(62.4)		
**Social activities**					44.066	< 0.001
Yes	Yes = 0	632(5.8)	374(7.3)	258(4.4)		
No	No = 1	10,348(94.2)	4720(92.7)	5628(95.6)		
**Sleep time(h)/day**	Continuous measurement	7.37(2.41)	7.75(2.14)	7.04(2.58)	15.967	< 0.001
**Smoking**					20.713	< 0.001
No	No = 0	9234(84.1)	4197(82.4)	5037(85.6)		
Yes	Yes = 1	1746(15.9)	897(17.6)	849(14.4)		
**Drinking**					79.451	< 0.001
No	No = 0	7264(66.2)	4156(81.6)	5162(87.7)		
Yes	Yes = 1	3716(33.8)	938(18.4)	724(12.3)		
**Exercises**					313.808	< 0.001
No	No = 0	7264(66.2)	2932(57.6)	4332(73.6)		
Yes	Yes = 1	3716(33.8)	2162(42.4)	1554(26.4)		
**Number of chronic diseases**					4.431	0.035
< 2	< 2 = 0	6661(60.7)	3144(61.7)	3517(59.8)		
≥ 2	≥ 2 = 1	4319(39.3)	1950(38.3)	2369(40.2)		
**Economic factors**						
**Sufficiency of living source**					196.795	< 0.001
Yes	Yes = 0	9539(86.9)	4673(91.7)	4866(82.7)		
No	No = 1	1441(13.1)	421(8.3)	1020(17.3)		
**Economic status**					197.641	< 0.001
Rich	Rich = 0	2259(20.6)	1345(26.4)	914(15.5)		
Poor	Poor = 1	8721(79.4)	3749(73.6)	4972(84.5)		
**Activity and Anxiety**						
**BADL**					56.637	< 0.001
Don’t need help	Don’t need help = 0	8994(81.9)	4324(84.9)	4670(79.3)		
Need help	Need help = 1	1986(18.1)	770(15.1)	1216(20.7)		
**IADL**					274.711	< 0.001
Independence	Independence = 0	4255(38.8)	2396(47.0)	1859(31.6)		
Dependence	Dependence = 1	6725(61.2)	2698(53.0)	4027(68.4)		
**Anxiety(GAD-7)**					765.024	< 0.001
No anxiety	No anxiety = 0	9700(88.3)	4964(97.4)	4736(80.5)		
Anxiety	Anxiety = 1	1280(11.7)	130(2.6)	1150(19.5)		

Note: p-values from chi-square tests or t-tests. Chi-square tests: 0 cells (0.0%) have expected count less than 5. BADL: The basic activities of daily living. IADL: The instrumental activities of daily living. depressive symptoms: The Center for Epidemiological Studies Depression Scale (CES-D-10). anxiety(GAD-7): The anxiety disorder scale

### Statistical analysis

Data were first analyzed by SPSS25.0 (IBM Corporation, NY, USA). The demographic characteristics were used as numbers and percentages or mean ± SD to express. Differences between groups were assessed by the Chi-square test (categorical data) and the t-test (continuous data). The association between living alone or not and CES-D-10 was then estimated using binary logistic regression with adjustment for confounding factors. In addition, GraphPad Prism 8 was used to draw the forest plots. Finally, Structural equation software Mplus 8.3 (Muthén & Muthén, Los Angeles, CA, USA) [51] was used to test the mediation effect between living alone or not and depressive symptoms with mediating variables. The statistical significance level was *P* < 0.05.

## Results

### Participants

Of the 15,874 participants in the CLHLS 2018 wave, 574 were living in a nursing institution, 326 were missing values of independent variables. Meanwhile, in this article we defined living alone, which strictly means living by oneself but with no other household members. And we defined living with household members (not living alone), which strictly means living with somebody else. Therefore, 401 ambiguous values of living alone and 255 ambiguous values of not living alone were also excluded. And also, 2974 participants were excluded, which were samples with missing values(> 5%) or responses of “I don’t know/unable to answer” or extreme values. Finally, the final analysis was restricted to 10,980 individuals older than 65 years. (Fig. 1).

### Basic characteristics and univariate analysis

As shown in Table 1, a total of 10,980 participants were selected for this study. The mean age was 83.63 ± 10.85 years old, 5134 (46.8%) males and 5846 (53.2%) females. 53.61% of the participants had depressive symptoms. Univariate analysis showed that gender, age, residence, marital status, living arrangements, self-reported health, sleep quality, social activities, sleep time (h)/day, smoking, drinking, exercises, number of chronic diseases, living source, economic status, BADL, IADL and anxiety had a statistically significant effect on depressive symptoms (*P*<0.05). The detailed data was presented in Table 1.

### Association between living alone or not and depressive symptoms

**Fig. 2:**
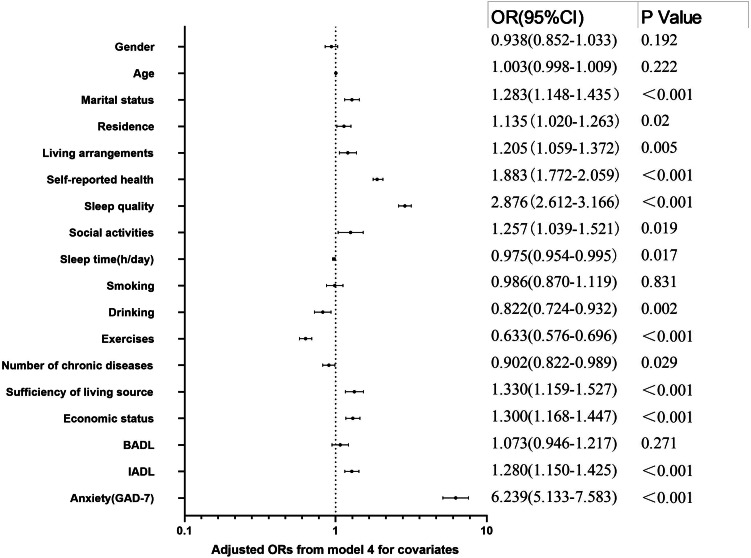
Adjusted ORs from Model 4. CI: Confidence Interval. OR: Odds Ratio

A hierarchical multiple model of the binary logistic regression was used to estimate the association between living alone or not and depressive symptoms [52]. As shown in Table 2, Model 1 explored the association between living alone or not and depressive symptoms when adjusting for sociodemographic information about gender, age, marital status and residence. Compared with those who did not live alone, those who lived alone had a significantly increased risk of depressive symptoms (OR = 1.26, 95% CI: 1.12, 1.41) (Table 2, Supplementary Table 1). Model 2 was shown when adjusting for gender, age, marital status, residence, self-reported health, sleep quality, social activities, sleep time and smoking, drinking, exercises and number of chronic diseases, participants who lived alone had a significantly increased risk of depressive symptoms (OR = 1.20, 95% CI: 1.06, 1.36) (Table 2, Supplementary Table 2). Further adjusting for gender, age, marital status, residence, self-reported health, sleep quality, social activities, sleep time and smoking, drinking, exercises and number of chronic diseases, sufficiency of living source, economic status, BADL, IADL and anxiety, participants who lived alone had a significantly increased risk of depressive symptoms (Model 3 and Model 4) (Table 2, Supplementary Tables 3 and Supplementary Table 4). In the multivariable-adjusted model (Model 4), participants who lived alone had an increased risk of depressive symptoms (OR = 1.21, 95% CI: 1.06, 1.37) (Table 2). These results suggested that living arrangements were an essential predictor for depressive symptoms in older adults. In addition, the forest plot of Model 4 was shown in Fig. 2. The forest plot indicated the following factors may be likely facilitators for depressive symptoms in older adults: marital status, sleep quality, living arrangements, self-reported health, social activities, sufficiency of living source, economic status, IADL and anxiety.

**Table 2:** Binary logistic regression to estimate the relationship between living alone or not and depressive symptoms

Characteristics	Model 1	Model 2	Model 3	Model 4
	*OR (95%CI)*	*OR (95%CI)*	*OR (95%CI)*	*OR (95%CI)*
Living arrangements				
Not living alone	1.00(reference)	1.00(reference)	1.00(reference)	1.00(reference)
Living alone	1.26^***^(1.12,1.41)	1.20^**^(1.06,1.36)	1.18^**^(1.04,1.34)	1.21^**^(1.06,1.37)

*P < 0.05, **P < 0.01, and ***P < 0.001. OR: Odds Ratio. 95%CI: 95% Confidence Interval

### The parallel mediation effect model

**Fig. 3:**
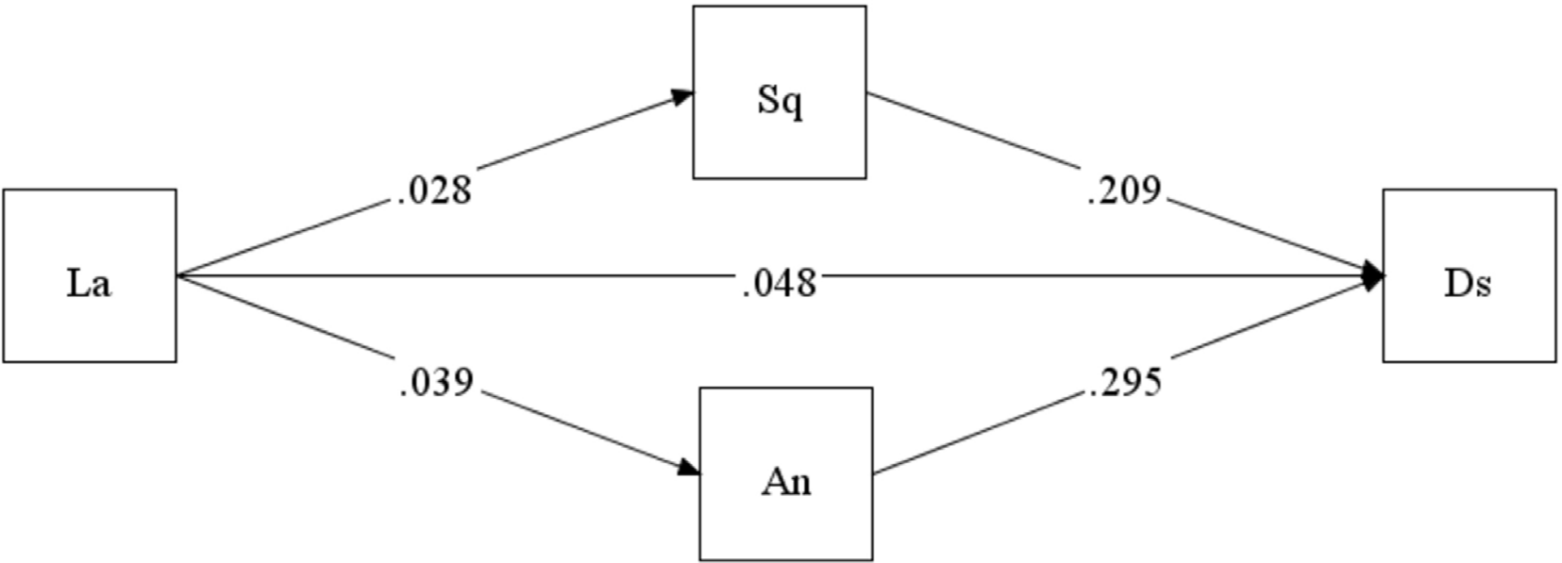
Figure of the parallel mediation effect model. La: living arrangements. Ds: depressive symptoms(CES-D-10). Sq: sleep quality. An: anxiety(GAD-7)

A parallel mediation model was used to test the likely mediation effect on the relationship between living alone or not and depressive symptoms. So a bootstrap test was carried out to verify the mediating effect of sleep quality and anxiety (Fig. 3). The results indicated that the mediating effect of sleep quality and anxiety on the relationship between living alone or not and depressive symptoms was significant. The total effect of living arrangements on depressive symptoms was significant (β = 0.089, 95% CI [0.065, 0.116], *P* < 0.001). The direct effect of living arrangements on depressive symptoms was significant (β = 0.048, 95% CI [0.031, 0.065], *P* < 0.001), accounting for 53.93% of the total effect. The indirect effect of living arrangements on depressive symptoms through sleep quality was significant (β = 0.008, 95% CI [0.003, 0.014], *P* < 0.01), accounting for 9% of the total effect. The indirect effect of living arrangements on depressive symptoms through anxiety was significant (β = 0.015, 95% CI [0.008, 0.024], *P* < 0.001), accounting for 17.3% of the total effect. Meanwhile, there was no significant difference in the mediating effect between sleep quality and anxiety (*P* = 0.091). The results demonstrated that both sleep quality and anxiety partially mediated the association between living alone or not and depressive symptoms. The detailed data was shown in Table 3

**Table 3:** Standardized effects for the parallel mediation model

Model pathways	Estimate	S.E.	95% CI		P
			Lower	Upper	
Direct La→Ds	0.048	0.009	0.031	0.065	<0.001
Indirect effect 1 La→Sq→Ds	0.008	0.003	0.003	0.014	0.007
Indirect effect 2 La→An→Ds	0.015	0.004	0.008	0.024	<0.001
Total effect La→Ds	0.089	0.013	0.065	0.116	<0.001
Indirect effect 1/Total	0.090	0.033	0.030	0.163	0.007
Indirect effect 2/Total	0.173	0.044	0.093	0.259	<0.001
DIFF(Indirect effect 1-Indirect effect 2)	−0.007	0.004	−0.016	0.001	0.091

Note: La: living arrangements. Ds: depressive symptoms(CES-D-10). Sq: sleep quality. An: anxiety(GAD-7).

## Discussion

This study aimed to investigate the relationship between living arrangements (living alone or not) and depressive symptoms in older adults in China. Meanwhile, the possible effects and mediating roles of sleep quality and anxiety in this relationship were also estimated. Consistent with the hypothesis, people who lived alone were more likely to be depressed. Sleep quality and anxiety might act as a parallel mediating role between the relationship between living alone or not and depressive symptoms.

Negative mental states in older adults, such as depressive symptoms, have been a focus of researchers. Many factors could cause depressive symptoms in older adults such as loneliness [53, 54], health and social–physical environment [55], living alone [56], chronic diseases [57], etc. This study found that living alone was one of the risk factors for depressive symptoms. This was in accordance with previous evidence [58–60]. Meanwhile, the current study also showed that participants who lived alone had higher rates of depressive symptoms than those not living alone. Likewise, previous studies have linked social isolation to an increased risk of elevated depressive symptoms in older adults in Japan [61]. Similarly, studies have also shown that those without a spouse or living alone were more likely to suffer from depressive symptoms [62]. These all pointed to the need for older adults living alone should be noticed and cared for to improve their quality of life.

Binary logistic regression analysis showed that after adjusting for confounding factors, the relationship between living alone or not and depressive symptoms was still significant. This strongly suggested that living alone or not could be associated with depressive symptoms. By comparing Model 4, we considered that sleep quality and anxiety might have some mediating effect. So we further analyzed the possible mediating roles.

The mediation analysis showed that sleep quality was positively related to living alone or not and depressive symptoms, and partially mediated the effect of living alone or not on depressive symptoms. The older adults living alone had poorer sleep quality and were more prone to have depressive symptoms. It might be that loneliness is more pronounced in individuals who live alone which may lead to sleep disturbance and thus lead to possible depressive symptoms [63, 64]. This may be reflected in the release of dopamine in the brain, which has been shown to play a role in sleep regulation and emotion regulation [65]. While sleep disturbance may affect emotional functioning [66]. Meanwhile, research has shown that the dopamine regulatory system may underlie the pathophysiology of depression [67]. Therefore, effectively adjusting the sleep quality of older adults living alone can partially prevent the occurrence of depressive symptoms. This is similar to previous study which showed that depressive symptoms could be improved by improving sleep through physical exercise [68]. In addition, studies have shown that improving sleep quality could improve depressive symptoms and quality of life [33, 69].

The mediation analysis also showed that anxiety was positively related to living alone or not and depressive symptoms, and partially mediated the effect of living alone or not on depressive symptoms. The older adults living alone with obvious anxiety are more likely to have depressive symptoms. Older adults who live alone commonly experience feelings of loneliness [70], which can contribute to the development of anxiety [38]. And the appearance of anxiety may be associated with specific brain neurotransmitter mechanisms [71]. As two mental disorders, anxiety and depression often co-occur, but research also suggested that anxiety typically precedes depression [72]. Therefore, it is possible that anxiety serves as a risk factor in the association between living alone and depression. Related studies have also demonstrated that depression and anxiety as a role of Mediating effect on the relationship between loneliness and cognitive function [73]. In addition, outdoor activities were the moderation between living alone or not and depressive symptoms in older adults [59]. Also, research indicated that subjective physical health, resilience and social support were the mediators between loneliness and depression in older women [74]. Nevertheless, through a rigorous literature search, there was no relevant literature examining the parallel mediating role of sleep quality and anxiety in the relationship between living alone or not and depressive symptoms. This study provided evidence that sleep quality and anxiety are mediating factors in the association between living alone or not and depressive symptoms in older adults.

Therefore, older adults living alone should exercise more properly to improve their sleep quality. Also, they should engage in more social activities to relieve anxiety and loneliness. How to improve the sleep quality and anxiety of older adults living alone was also an issue that community workers needed to focus on and consider. First, mental health advocacy could be undertaken to improve the mental health of older adults living alone. Second, regular mental health counselling should be provided to older adults living alone to reduce the occurrence and development of negative mental states. Third, increasing companionship has a modest effect on improving the mental health of older adults living alone.

Our study had some strengths. First, this was the first study to identify sleep quality and anxiety as parallel mediators between living alone or not and depressive symptoms. Second, adjusting for a wide range of covariates allowed us to incorporate major potential confounders and better account for the association between living alone or not and depressive symptoms. Also, the study reflected some potential limitations. First of all, the data was a cross-sectional investigation, so the conclusion might only be explained by statistics [75]. Longitudinal data may be needed to explore this relationship further. Second, depressive symptoms in older adults might be associated with various factors, and our research might reflect in a few aspects. This study mainly considered psychosocial factors, so variables such as healthcare and dietary habits were not included. Third, this study used a screening instrument for depression. Therefore, the findings reflected only the relationship between living alone or not and depressive symptoms but not for depression. Fourth, variables were derived from self-report which might have led to bias [76]. Thus, more relevant studies are needed to provide evidence for the present findings.

## Conclusions

The study investigated the association of living alone or not with depressive symptoms in older adults. After adjusting for many covariates, older adults who lived alone still had higher depressive symptoms. While the results of the parallel mediation analysis indicated that sleep quality and anxiety had a parallel mediating effect. Sleep quality and anxiety might explain the pathway from living arrangements to depressive symptoms. This might suggest a different approach to intervention strategies for depressive symptoms in older adults living alone. Older adults who lived alone with worse sleep quality and more pronounced anxiety were positively associated with higher depressive symptoms. Therefore, appropriate interventions should be implemented to reduce depressive symptoms in older adults who lived alone. Older adults who lived alone should be encouraged to engage in social activities that improve sleep quality, relieve anxiety, and improve feelings of loneliness caused by living alone.

### Electronic supplementary material

Below is the link to the electronic supplementary material.

**Supplementary Table 1**. Binary logistic regression to estimate the relationship between living alone or not and depressive symptoms(Model 1). **Supplementary Table 2**. Binary logistic regression to estimate the relationship between living alone or not and depressive symptoms(Model 2). **Supplementary Table 3**. Binary logistic regression to estimate the relationship between living alone or not and depressive symptoms(Model 3). **Supplementary Table 4**. Binary logistic regression to estimate the relationship between living alone or not and depressive symptoms(Model 4).

## Data Availability

The CLHLS data were acquired from Peking University Open Research Data and are available at https://opendata.pku.edu.cn/dataset.xhtml?persistentId=doi:10.18170/DVN/WBO7LK. Permission for data use in this study was given by the CLHLS.

## Abbreviations

CLHLS: Chinese Longitudinal Healthy Longevity Survey
CES-D-10: Center for Epidemiological Studies Depression Scale
GAD-7: Anxiety Disorder Scale
BADL: Basic Activities of Daily Living
IADL: Instrumental Activities of Daily Living
