# Supplementary material for: The relationship between living alone or not and depressive symptoms in older adults: a parallel mediation effect of sleep quality and anxiety
Source: BMC Geriatr. 2023 Aug 22;23:506. doi: 10.1186/s12877-023-04161-0 (PMC10463962; doi:10.1186/s12877-023-04161-0)
Supplement: Supplementary file 1 — Supplementary Table 1. Binary logistic regression to estimate the relationship between living alone or not and depressive symptoms(Model 1). Supplementary Table 2. Binary logistic regression to estimate the relationship between living alone or not and depressive symptoms(Model 2). Supplementary Table 3. Binary logistic regression to estimate the relationship between living alone or not and depressive symptoms(Model 3). Supplementary Table 4. Binary logistic regression to estimate the relationship between living alone or not and depressive symptoms(Model 4). [file 12877_2023_4161_MOESM1_ESM.docx]

**Supplementary Table 1**. Binary logistic regression to estimate the relationship between living alone or not and depressive symptoms(Model 1)

| **Characteristics** | **B** | **S.E.** | **Wald** | ***P*** | **OR** | **(95%CI)** |
| --- | --- | --- | --- | --- | --- | --- |
| Sociodemographic | | | | | | |
| Gender | 0.250 | 0.041 | 37.892 | ＜0.001 | 1.284 | 1.186-1.391 |
| Age(years) | 0.010 | 0.002 | 22.377 | ＜0.001 | 1.010 | 1.006-1.014 |
| Marital status | 0.188 | 0.051 | 13.715 | ＜0.001 | 1.207 | 1.093-1.333 |
| Residence | 0.334 | 0.046 | 52.402 | ＜0.001 | 1.396 | 1.275-1.528 |
| **Living arrangements** | 0.230 | 0.059 | 15.417 | **＜0.001** | **1.259** | **1.122-1.412** |
| Constant | -1.702 | 0.203 | 70.359 | 0.000 | 0.182 |  |

OR: Odds Ratio. 95%CI: 95% Confidence Interval. Model 1: adjusted for gender, age, marital status and residence.

**Supplementary Table 2**. Binary logistic regression to estimate the relationship between living alone or not and depressive symptoms(Model 2)

| **Characteristics** | **B** | **S.E.** | **Wald** | ***P*** | **OR** | **(95%CI)** |
| --- | --- | --- | --- | --- | --- | --- |
| Sociodemographic | | | | | | |
| Gender | 0.011 | 0.048 | 0.054 | 0.817 | 1.011 | 0.921-1.110 |
| Age(years) | 0.008 | 0.002 | 11.858 | 0.001 | 1.008 | 1.004-1.013 |
| Marital status | 0.268 | 0.055 | 23.423 | ＜0.001 | 1.307 | 1.173-1.457 |
| Residence | 0.206 | 0.053 | 15.198 | ＜0.001 | 1.228 | 1.108-1.362 |
| **Living arrangements** | 0.185 | 0.064 | 8.456 | **0.004** | **1.204** | **1.062-1.364** |
| Health-related | | | | | | |
| Self-reported health | 0.785 | 0.044 | 322.118 | ＜0.001 | 2.192 | 2.012-2.388 |
| Sleep quality | 1.145 | 0.048 | 570.412 | ＜0.001 | 3.144 | 2.862-3.454 |
| Social activities | 0.224 | 0.094 | 5.663 | 0.017 | 1.251 | 1.040-1.504 |
| Sleep time(h)/day | -0.033 | 0.011 | 9.946 | 0.002 | 0.967 | 0.948-0.988 |
| Smoking | 0.004 | 0.063 | 0.004 | 0.952 | 1.004 | 0.888-1.135 |
| Drinking | -0.217 | 0.063 | 11.963 | 0.001 | 0.805 | 0.712-0.910 |
| Exercises | -0.501 | 0.047 | 115.717 | ＜0.001 | 0.606 | 0.553-0.664 |
| Chronic diseases | -0.062 | 0.045 | 1.855 | 0.173 | 0.940 | 0.860-1.028 |
| Constant | -1.725 | 0.256 | 45.415 | 0.000 | 0.178 |  |

OR: Odds Ratio. 95%CI: 95% Confidence Interval. Model 2: adjusted for gender, age, marital status, residence, self-reported health, sleep quality, social activities, sleep time and smoking, drinking, exercises and number of chronic diseases.

**Supplementary Table 3**. Binary logistic regression to estimate the relationship between living alone or not and depressive symptoms(Model 3)

| **Characteristics** | **B** | **S.E.** | **Wald** | ***P*** | **OR** | **(95%CI)** |
| --- | --- | --- | --- | --- | --- | --- |
| Sociodemographic | | | | | | |
| Gender | 0.004 | 0.048 | 0.005 | 0.941 | 1.004 | 0.914-1.102 |
| Age(years) | 0.010 | 0.002 | 15.913 | ＜0.001 | 1.010 | 1.005-1.014 |
| Marital status | 0.267 | 0.056 | 23.047 | ＜0.001 | 1.306 | 1.171-1.456 |
| Residence | 0.153 | 0.053 | 8.283 | 0.004 | 1.165 | 1.050-1.294 |
| **Living arrangements** | 0.167 | 0.064 | 6.809 | **0.009** | **1.182** | **1.042-1.340** |
| Health-related | | | | | | |
| Self-reported health | 0.718 | 0.044 | 261.763 | ＜0.001 | 2.051 | 1.880-2.238 |
| Sleep quality | 1.113 | 0.048 | 533.578 | ＜0.001 | 3.045 | 2.770-3.347 |
| Social activities | 0.207 | 0.094 | 4.840 | 0.028 | 1.230 | 1.023-1.480 |
| Sleep time(h)/day | -0.034 | 0.011 | 10.143 | 0.001 | 0.967 | 0.947-0.987 |
| Smoking | -0.013 | 0.063 | 0.045 | 0.831 | 0.987 | 0.872-1.116 |
| Drinking | -0.213 | 0.063 | 11.462 | 0.001 | 0.808 | 0.714-0.914 |
| Exercises | -0.473 | 0.047 | 102.174 | ＜0.001 | 0.623 | 0.568-0.683 |
| Chronic diseases | -0.044 | 0.046 | 0.918 | 0.338 | 0.957 | 0.875-1.047 |
| Economic factors | | | | | | |
| Sufficiency of living source | 0.423 | 0.068 | 38.779 | ＜0.001 | 1.527 | 1.336-1.744 |
| Economic status | 0.292 | 0.054 | 29.524 | ＜0.001 | 1.339 | 1.205-1.487 |
| Constant | -1.996 | 0.260 | 59.073 | 0.000 | 0.136 |  |

OR: Odds Ratio. 95%CI: 95% Confidence Interval. Model 3: adjusted for gender, age, marital status, residence, self-reported health, sleep quality, social activities, sleep time and smoking, drinking, exercises and number of chronic diseases, sufficiency of living source, economic status.

**Supplementary Table 4**. Binary logistic regression to estimate the relationship between living alone or not and depressive symptoms(Model 4)

| **Characteristics** | **B** | **S.E.** | **Wald** | ***P*** | **OR** | **(95%CI)** |
| --- | --- | --- | --- | --- | --- | --- |
| Sociodemographic | | | | | | |
| Gender | -0.064 | 0.049 | 1.704 | 0.192 | 0.938 | 0.852-1.033 |
| Age(years) | 0.003 | 0.003 | 1.489 | 0.222 | 1.003 | 0.998-1.009 |
| Marital status | 0.250 | 0.057 | 19.208 | ＜0.001 | 1.283 | 1.148-1.435 |
| Residence | 0.127 | 0.055 | 5.382 | 0.020 | 1.135 | 1.020-1.263 |
| **Living arrangements** | 0.187 | 0.066 | 8.005 | **0.005** | **1.205** | **1.059-1.372** |
| Health-related | | | | | | |
| Self-reported health | 0.633 | 0.046 | 192.442 | ＜0.001 | 1.883 | 1.722-2.059 |
| Sleep quality | 1.056 | 0.049 | 463.194 | ＜0.001 | 2.876 | 2.612-3.166 |
| Social activities | 0.229 | 0.097 | 5.515 | 0.019 | 1.257 | 1.039-1.521 |
| Sleep time(h)/day | -0.026 | 0.011 | 5.701 | 0.017 | 0.975 | 0.954-0.995 |
| Smoking | -0.014 | 0.064 | 0.046 | 0.831 | 0.986 | 0.870-1.119 |
| Drinking | -0.196 | 0.064 | 9.281 | 0.002 | 0.822 | 0.724-0.932 |
| Exercises | -0.457 | 0.048 | 89.388 | ＜0.001 | 0.633 | 0.576-0.696 |
| Chronic diseases | -0.103 | 0.047 | 4.776 | 0.029 | 0.902 | 0.822-0.989 |
| Economic factors | | | | | | |
| Sufficiency of living source | 0.285 | 0.070 | 38.779 | ＜0.001 | 1.330 | 1.159-1.527 |
| Economic status | 0.262 | 0.055 | 29.524 | ＜0.001 | 1.300 | 1.168-1.447 |
| ADL and Anxiety | | | | | | |
| BADL | 0.071 | 0.064 | 1.213 | 0.271 | 1.073 | 0.946-1.217 |
| IADL | 0.247 | 0.055 | 20.327 | ＜0.001 | 1.280 | 1.150-1.425 |
| Anxiety(GAD-7) | 1.831 | 0.100 | 338.105 | ＜0.001 | 6.239 | 5.133-7.583 |
| Constant | -1.637 | 0.282 | 33.695 | 0.000 | 0.195 |  |

OR: Odds Ratio. 95%CI: 95% Confidence Interval. Model 4: gender, age, marital status, residence, self-reported health, sleep quality, social activities, sleep time and smoking, drinking, exercises and number of chronic diseases, sufficiency of living source, economic status, BADL, IADL and anxiety.
